# The effect of health facility ownership on perceived healthcare quality: evidence from Ghana

**DOI:** 10.1007/s10754-024-09385-0

**Published:** 2024-09-16

**Authors:** Alex Bawuah, Simon Appleton, Yang Li

**Affiliations:** 1 Present Address: Health Economics Unit, Aberystwyth Business School, Aberystwyth University, Aberystwyth, UK; 2 School of Economics, University of Nottingham, Nottingham, UK; 3 School of Economics, University of Nottingham Ningbo China, Ningbo, China

**Keywords:** Quality, Healthcare, Public facility, Private facility, Ghana, I10, I11

## Abstract

Whether private healthcare providers should be encouraged over public providers remains unclear. On the one hand, because private providers are profit-driven, they are more motivated to compete for demand by enhancing quality if demand is elastic. However, because they are more motivated to maximize revenue, they may sacrifice quality to maximize profit. A crucial factor in determining whether private providers should be encouraged is the extent to which their quality exceeds or falls short of that of the public provider. This study, therefore, investigates whether the public and private differ in providing quality healthcare services using the 2014 Ghana Demographic and Health Survey. Our measure of healthcare quality is based on patient satisfaction level with nine healthcare services (cleanliness, waiting time, comfort and safety, consultation time, privacy, listening, explanation, treatment advice and confidentiality) provided by public and private healthcare facilities. We applied an instrumental variable approach to account for endogeneity issues related to the patient’s choice of healthcare provider. We find that private facility users have a higher probability of being very satisfied with “waiting time”, “consultation time”, “listening”, “cleanliness”, “comfort and safety”, “confidentiality”, and “privacy” than public users, thus suggesting that private facilities provide better service than public. We thus recommend encouraging the private sector to enter the healthcare market. We also find that failing to account for endogeneity in provider choice when estimating the effect of healthcare facility ownership on healthcare service quality underestimates the effects.

## Introduction

The disparity between the demand and supply of healthcare services and the quality of healthcare services is a major issue for many developing countries. This is because the public health sector’s resources alone are often insufficient to provide universal healthcare, improve healthcare quality, and expand access. In light of this, a resolution was passed by the World Health Assembly calling on countries to “constructively engage the private sector in providing essential healthcare services” based on evidence that private healthcare providers play a major role in healthcare delivery across the world to achieve universal health coverage (WHO, 2010). However, achieving universal health coverage will become an empty vessel if the quality of healthcare services is poor; hence, governments are advised to ensure that universal healthcare coverage is associated with quality healthcare service (The National Academies of Sciences Engineering & Medicine, 2018). As such, a crucial factor in determining whether entry of private healthcare providers should be encouraged is the extent to which their quality exceeds or falls short of that of the public provider. On the one hand, because private providers are profit-oriented, they have a stronger incentive to compete for demand by raising quality if demand is sufficiently elastic (Moscone et al., 2020). On the other hand, they might skimp on quality to obtain a higher profit because they have a stronger incentive to maximise profits. (Moscone et al., 2020). This study investigates whether there are differences in healthcare quality (where quality is measured by user satisfaction levels) between public and private healthcare providers using data from Ghana.

Many studies have examined the effect of health facility ownership on healthcare quality; however, there seem to be no clear-cut conclusions on the subject matter as the existing empirical literature provides mixed results. Findings from some studies suggest that public (government) healthcare facilities provide better quality healthcare than private facilities (Chin et al., 2020; Lien et al., 2008; Onwujekwe et al., 2009; Wang et al., 2017). In construct, the findings from other studies suggest that private healthcare facilities provide better quality (Alumran et al., 2020; Fall, 2017; Jensen et al., 2009; Milcent, 2005). However, Pérotina et al. (2013), Moscelli et al. (2018), and Moscone et al. (2020) provide evidence which suggests no significant differences in the quality of healthcare between private and public facilities. Moreover, Swain (2019) found that public and private facilities perform better in certain quality dimensions than others.

Ghana’s healthcare system is characterised by fierce competition between the private and public sectors, which has pushed service quality to the forefront of national healthcare policies (Atinga et al., 2011). However, studies on the effect of healthcare facility ownership on healthcare have generally suggested that private facilities provide better healthcare services. Anabila et al. (2019) found that patients perceive that private hospitals’ service quality is better than the public. Adongo et al. (2021) also showed that private hospitals offered patients better healthcare services compared to the public. Similarly, Owusu Kwateng et al. (2017) found that patients’ perception of health service quality in private hospitals was higher than in public hospitals. Alhassan et al. (2016) also found that quality indicators, including drug availability, respectfulness of health staff and client waiting times, were perceived to be moderately better by patients who visit private health facilities than public.

While we acknowledge that the studies from Ghana have contributed to the body of knowledge in this area in the Ghanaian context, they have suffered from some weaknesses. First, none of the studies accounted for issues of endogenous selection with provider choice. The consequence of not accounting for endogenous selection is that the estimated effect of health facility ownership on quality may be biased (Lien et al., 2008; Moscelli et al., 2018; Moscone et al., 2020; Pérotina et al., 2013). We address this issue using an IV approach to account for endogenous selection associated with provider choice. To the best of our knowledge, this is the first study to examine the quality of healthcare between private and public providers in Ghana that accounts for issues of endogenous selection with provider choice.

Second, our measures/indicators of health service quality differ from the previous studies. We measure healthcare quality using patient satisfaction with nine dimensions of healthcare services, including waiting time, consultation time, explanation, listening, treatment advice, cleanliness, comfort and safety, privacy, and confidentiality. However, previous studies have often used satisfaction with five dimensions of healthcare services as indicators of healthcare quality, including empathy, reliability, responsiveness, assurance, and tangibility (Adongo et al., 2021; Alhassan & Nketiah-Amponsah, 2016; Anabila et al., 2019; Owusu Kwateng et al., 2017). Thus, our study provides evidence of other dimensions of health services which have not yet been explored in the literature.

Finally, the previous studies used small-scale datasets (datasets restricted to specific geographical locations–Ashanti, Western, and Greater Accra regions) (Adongo et al., 2021; Alhassan & Nketiah-Amponsah, 2016; Anabila et al., 2019; Owusu Kwateng et al., 2017), hence, limiting their findings’ generalizability. We address this issue by using nationally representative data in our analysis. Hence, the findings of our study give a general representation of the quality of healthcare between private and public healthcare providers in Ghana.

## Methods

### Study setting and data source

Ghana is a lower-middle-income country in West Africa. Its current population size is about 30.8 million (Ghana Statistical Service, 2023). Ghana has 16 regions serving as its primary administrative sub-divisions.

This study used data from the 2014 Ghana Demographic and Health Survey (GDHS). The 2014 GDHS is a nationally representative survey that collects data on demographic and health indicators including fertility, family planning, maternal and child health, adult and childhood mortality, women’s empowerment, domestic violence, malaria, HIV/AIDS and other sexually transmitted infections (STIs), and other health-related issues from men and women aged 15–59 (GSS; GHS; ICF International, 2015).

### Sampling design and study sample

The 2014 GDHS employed a two-stage sampling process. In the first stage, sample points (clusters) consisting of enumeration areas (EAs) were selected (GSS; GHS; ICF International, 2015). Four hundred and twenty-seven (427) clusters were chosen, including 211 in rural and 216 in urban areas. A household listing operation was carried out in all the selected EAs. The second stage involved the systematic sampling of households from the selected households. Thirty households were chosen from each cluster to constitute a sample size of 12,810 households. The 30 households were chosen in a way that gave each one an equal chance of being selected (GSS; GHS; ICF International, 2015).

The sample for this study includes men and women who were interviewed in the selected households. Overall, 9396 women aged 15–49 years and 4,388 men aged 15–59 were successfully interviewed (GSS; GHS; ICF International, 2015). After cleaning the data and dropping all missing observations (for this study), we ended up with a sample size of 4324. This is made up of individuals who had visited a healthcare facility six months before the survey.

### Estimation strategy

We estimate the effect of health facility ownership on the quality of healthcare using the following estimation technique.

1 $${y}_{i}^{*}={\beta }_{1}{X}_{i}+{\beta }_{2}{M}_{i}+{e}_{1}$$

where ${y}_{i}^{*}$ is a latent (unobserved) response for the $i$th individual (the response is the individual’s level of satisfaction with healthcare services). ${X}_{i}$ is a dummy variable taking a value of “1” if the healthcare facility visited by the $i$th individual is private and “0” otherwise. ${M}_{i}$ denotes characteristics of the $i$th individual (age, gender, level of education, etc.) and ${e}_{1}$ is the error term. ${\beta }_{1}$ is the parameter of interest.

Let ${y}_{i}$ be the observed response for the $i$th individual, which takes on the values $j$ = $\{\text{1,2},\dots ,m\}$. Let ${\alpha }_{1}<{\alpha }_{2}<\dots <{\alpha }_{m}$ be a threshold (cut points) such that

2 $${y}_{i}=\left\{\begin{array}{c}1 if {y}_{i}^{*} \le {\alpha }_{1} \\ 2 if {\alpha }_{1}<{y}_{i}^{*} \le {\alpha }_{2} \\ . . . \\ . . . \\ . . . \\ m if {\alpha }_{m-1}<{y}_{i}^{*}\end{array}\right.$$

It then follows that $P\left( {y_{i} = j} \right) = P(\alpha_{m - 1} < y_{i}^{*} \le \alpha_{1} )$.

One major concern in estimating Eq. (1) is that other unobserved factors may influence an individual’s decision to visit a private healthcare facility. If that is the case, then estimating Eq. (1) will produce biased estimates. We, therefore, employ the instrumental variable (IV) approach to address this issue. The IV approach has been used in similar studies to address this kind of issue (Lien et al., 2008; Moscelli et al., 2018; Moscone et al., 2020).

3 $$X_{i} = 1\left( {\gamma_{1} M_{i} + \gamma_{2} Z + e_{2} > 0} \right)$$

where $Z$ is an instrumental variable and $\gamma$ are parameters to be estimated. The instrumental variable must meet two requirements; (i) it should be correlated with the endogenous explanatory regressor, and (ii) it should be uncorrelated with the outcome variable except through their effect on the endogenous variable (Bowden & Darrell, 1990).

We can, thus, estimate the effect of health facility ownership on the quality of healthcare in two stages simultaneously (where Eq. (3) is the first stage and Eq. (1) is the second stage). This was done using the “*eoprobit*” command in Stata. The command supports the estimation of ordered probit regression models with binary endogenous covariates (StataCorp, 2019, 103–130).

### Measuring healthcare quality

Studies on the effect of healthcare facility ownership on healthcare quality have used two main indicators in measuring quality healthcare, namely, subjective measures and objective measures. In this study, we use a subjective measure as an indicator of healthcare quality, given the nature of our data (survey data). The use of subjective measures for determining healthcare quality is widely accepted (McDowell, 2006) and has been applied extensively in many studies relating to the quality of healthcare (Anabila et al., 2019; Chin et al., 2020; Gutacker et al., 2016; Pérotina et al., 2013). Gronross (1988) argues that the best people to assess the quality of a product are the consumers. Since patients are customers of healthcare services, they will be in the best position to determine the quality of healthcare they receive. Furthermore, Avdic et al. (2019) provide evidence suggesting that patients’ satisfaction with healthcare quality is an important complement to clinical indicators when choosing a healthcare provider. Moreover, studies have shown that subjective healthcare measures effectively predict health outcomes such as morbidity and mortality (Idler & Kasp, 1991; Kaplan & Camacho, 1983; Mossey & Shapiro, 1982). That is, the realities of healthcare are dominantly reflected in patients’ satisfaction ratings (Ware et al., 1983); therefore, consumers’ perceptions about healthcare quality may be used as a valid assessment of healthcare quality (Davies & Ware, 1988).

### Variables

The dependent variable is the individual’s level of satisfaction with nine healthcare services they received when they visited the healthcare facility. In the survey, the respondents were asked to rate their level of satisfaction with multiple healthcare services they received at the healthcare facilities they visited. The satisfaction levels were measured on a scale of 1 to 5 where “1 = very satisfied and 5 = very dissatisfied”. For this study, we recoded the satisfaction levels such that a higher number indicates higher satisfaction, thus “5 = very satisfied and 1 = very dissatisfied. We, thus, use the individual level of satisfaction with healthcare services as an indicator of healthcare quality following previous studies (Adongo et al., 2021; Fall, 2017; Pérotina et al., 2013). The variables include cleanliness, waiting time, comfort and safety, consultation time, privacy, listening, explanation, treatment advice and confidentiality (the definitions of these variables are provided in Table 1).

**Table 1:** Description and measurement of the variables for the study (2014 Ghana DHS data)

Variable name	Description and measurement
*Dependent variables*	
Cleanliness	Satisfaction with “the cleanliness of the facility”: 5 = “Very satisfied”, 4 = “Satisfied”, 3 = “Fairly satisfied”, 2 = “Not satisfied”, 1 = “Very dissatisfied”
Waiting time	Satisfaction with the “time to wait for your turn”: 5 = “Very satisfied”, 4 = “Satisfied”, 3 = “Fairly satisfied”, 2 = “Not satisfied”, 1 = “Very dissatisfied”
Comfort and Safety	Satisfaction with “comfort and safety while waiting”: 5 = “Very satisfied”, 4 = “Satisfied”, 3 = “Fairly satisfied”, 2 = “Not Satisfied”, 1 = “Very dissatisfied”
Consultation time	Satisfaction with “time spent in consulting/examination room”: 5 = “Very satisfied”, 4 = “Satisfied”, 3 = “Fairly satisfied”, 2 = “Not Satisfied”, 1 = “Very dissatisfied”
Privacy	Satisfaction with “privacy during examination”: 5 = “Very satisfied”, 4 = “Satisfied”, 3 = “Fairly satisfied”, 2 = “Not Satisfied”, 1 = “Very dissatisfied”
Listening	Satisfaction with the staff when they “listened to the you”: 5 = “Very satisfied”, 4 = “Satisfied”, 3 = “Fairly satisfied”, 2 = “Not Satisfied”, 1 = “Very dissatisfied”
Explanation	Satisfaction with the staff when they “explained what you wanted to you”: 5 = “Very satisfied”, 4 = “Satisfied”, 3 = “Fairly satisfied”, 2 = “Not Satisfied”, 1 = “Very dissatisfied”
Treatment advice	Satisfaction with the staff when they “gave advice and information on options for”: 5 = “Very satisfied”, 4 = “Satisfied”, 3 = “Fairly satisfied”, 2 = “Not Satisfied”, 1 = “Very dissatisfied”
Confidentiality	Satisfaction with “Confidentiality and protection of personal information.” 5 = “Very satisfied”, 4 = “Satisfied”, 3 = “Fairly satisfied”, 2 = “Not Satisfied”, 1 = “Very dissatisfied”
*Endogenous variable*	
Facility type	The type of healthcare facility the respondent visited: 1 = Private 0 = Public
Instrumental variable	
Facility’s location	Convenience of the facility’s location: 1 = “Very convenient”, 2 = “convenient”, 3 = “Fairly convenient”, 4 = “Not convenient”, 5 = “Very inconvenient”
*Control variables*	
Gender	Gender: 1 = Female, 0 = Male
Age	Age: 1 = 15–29 2 = 30–44 3 = 45–59
Marital Status	Individual’s marital status: 1 = Married, 0 = Otherwise
Education	Highest level of education: 1 = No education, 2 = Primary, 3 = Secondary, 4 = Higher
Employment	Employment status: 1 = Employed, 0 = Unemployed
Wealth	Wealth quintile: 1 = Poorest, 2 = Poorer, 3 = Middle, 4 = Rich, 5 = Richest
Residence	Place of residence: 1 = Rural, 0 = Urban
Patient type	Outpatient = 1, Inpatient = 0
Mode of payment	Cash = 1, 2 = Insurance, 3 = Co-payment (combination of insurance and cash), 4 = Others
Type of illness/services	1 = Family planning, 2 = Maternal Healthcare Services, 3 = Fever, 4 = Diarrhoea, 5 = HIV/AIDS/STI, 6 = High blood pressure, 7 = Ear/Nose/Throat infection, 8 = Diabetes, 9 = Eye infection, 10 = Chek-up/preventive care, 11 = Accident/injury, 12 = Child illness, 13 = Own illness, 14 = Others

Human Immunodeficiency Viruses (HIV), Acquired Immunodeficiency Syndrome (AIDS), Sexually Transmitted Infections (STI)

The independent variable of interest is the type of healthcare provider visited by the individual, with 1 = private healthcare facility and a 0 = public/government healthcare facility.

Based on previous studies, the distance from an individual’s residence to the nearest healthcare provider is the obvious candidate for instruments (Lien et al., 2008; Moscone et al., 2020). However, we do not have data on the distance from the individual’s residence to the nearest healthcare provider. Therefore, we use how convenient the healthcare facility’s location is from the respondent’s location as a proxy for the distance the individual has to travel to the healthcare facility. In the survey, the respondents were asked to rate how convenient the location of the healthcare facility was for them on a 5-point Likert scale from “very convenient” to “very inconvenient”, with “very convenient” implying short distance and “very inconvenient” as long distance. The idea is that individuals will prefer to use healthcare facilities whose locations are more convenient for them, all other things being equal.

We made two assumptions regarding our instrumental variable. First, the instrument (facility’s location) is uncorrelated with unobserved individual characteristics. This assumption may be invalid in the presence of spatial sorting, where individuals with poor health conditions migrate to live closer to the healthcare facilities (where access to healthcare will be more convenient). However, we do not think this is an issue within the Ghanaian context, where migration decision is often influenced by finding job opportunities and proximity to the workplace (Kwankye et al., 2009; Poku-Boansi & Adarkwa, 2016). Second, the healthcare facility’s location will influence people’s decision to use it; however, it will not influence the quality of healthcare services provided at the healthcare facilities. This assumption may not hold in a situation where individuals bypass facility “A” where they can conveniently get access to healthcare to use facility “B” where it is inconvenient to access healthcare because facility “B” offers better services than facility “A”. However, these instances do not often occur in Ghana since Ghana’s healthcare system is designed such that the first point of primary healthcare is at the local level health facilities where it is convenient for the people within that local or district to access healthcare. Patients are asked to use healthcare facilities from other places when the illness is beyond the primary healthcare provider. However, the type of illness/service listed by respondents in this study is not that which is beyond the capabilities of the primary healthcare facilities. Therefore, in this study, we assume that the convenience of the facility’s location may not have an impact on the perceived quality of healthcare services. Even if it does, the impact may not be significant.

The challenge with our instrumental variable is that we only have data on the facility the respondent used. However, we also need data on the convenience of the location of the facility, which the individual did not use. That is, if the individual had used an alternative provider, what would have been their response regarding the facility’s location? We address this challenge by using an ordered logistic regression imputation method to impute the data had the alternative provider been chosen (Raghunathan et al., 2001; Van Buuren, 2007, 2018). We use the ordered logistic regression imputation method because the variable of interest is ordinal. We applied the “*mi impute ologit”* command in Stata 17 to do the imputation (StataCorp, 2021, pp. 239–243).

The baseline equation used for the imputation is given as follows:

4 $$L_{j} = \beta Z + \varepsilon$$

Where $L$ represents the convenience of the location of the healthcare facility, $j$ is the ownership type of healthcare facility (public and private), and $Z$ denotes the vector of observed predictors of the convenience of the location of the healthcare facilities. $Z$ include the area of residence (rural/urban), district and administrative region of the respondents.

Patients have the liberty to choose their healthcare providers, and their choices may be affected by their morbidity. Moreover, private providers may also have stronger incentives to avoid more severe and consequently may appear to provide better quality if there is no adequate adjustment for case-mix. To control for different case-mix, we include a range of control variables (individual characteristics), including gender, age, marital status, level of education, wealth, employment status, residence, patient type, type of illness/services, and mode of payment. The names and definitions of all the variables for the study are listed in Table 1.

## Results

### Descriptive statistics

The descriptive statistics of the study participants are reported in Table 2. It shows that most respondents visited public healthcare facilities (81.6%), consistent with that of Awoke et al. (2017). Also, most respondents are females (83.72%), married (51.89%), employed (78.52%), and have secondary education (56.64%). Also, private facility users are usually aged between 30 and 44, whereas public users are often aged between 15 and 29. Furthermore, private facilities users are often in the richest wealth bracket (47.26%), whereas public users are often in the middle wealth bracket (22.47%). Most private users resided in urban areas (76.95%), whereas most public facilities users resided in rural areas (51.37%). In addition, most public users indicated that the facility’s location is “convenient” for them (50.12%), whereas the private users often indicated that the facility’s location is “very convenient” for them (45.66%).

**Table 2:** Descriptive summary of the sample (from the 2014 Ghana DHS individual data)

		Total N = 4,324	Private N = 796	Public N = 3,528
Variables	Categories	%	%	%
*Dependent variables*				
Cleanliness	Very satisfied	48.64	58.74	45.51
	Satisfied	42.83	34.36	45.46
	Fairly satisfied	7.07	5.78	7.47
	Dissatisfied	1.26	0.96	1.36
	Very dissatisfied	0.19	0.16	0.20
Waiting time	Very satisfied	26.17	37.33	22.71
	Satisfied	41.75	41.22	41.91
	Fairly satisfied	15.32	11.42	16.53
	Dissatisfied	11.05	6.95	12.32
	Very dissatisfied	5.44	3.07	6.17
	Not applicable	0.28	0.00	0.36
Comfort and safety	Very satisfied	38.13	48.64	34.86
	Satisfied	49.43	44.87	50.85
	Fairly satisfied	9.69	5.26	11.06
	Dissatisfied	2.27	0.65	2.78
	Very dissatisfied	0.48	0.58	0.44
Listening	Very satisfied	46.91	56.58	43.91
	Satisfied	44.09	38.03	45.97
	Fairly satisfied	6.72	3.96	7.58
	Dissatisfied	1.95	1.44	2.10
	Very dissatisfied	0.33	0.00	0.43
Consultation time	Very satisfied	29.29	41.15	25.61
	Satisfied	49.78	44.89	51.29
	Fairly satisfied	13.26	9.84	14.32
	Dissatisfied	4.43	2.03	5.18
	Very dissatisfied	2.00	1.45	2.17
	Not applicable	1.25	0.64	1.44
Privacy	Very satisfied	43.02	55.00	39.31
	Satisfied	44.53	37.72	46.64
	Fairly satisfied	8.94	5.02	10.16
	Dissatisfied	2.47	1.28	2.84
	Very dissatisfied	1.03	0.98	1.05
Explanation	Very satisfied	42.56	51.44	39.82
	Satisfied	43.26	36.80	45.26
	Fairly satisfied	9.62	7.00	10.43
	Dissatisfied	3.95	3.83	3.99
	Very dissatisfied	0.61	0.94	0.51
Treatment advice	Very satisfied	40.82	49.95	37.98
	Satisfied	42.64	36.76	44.46
	Fairly satisfied	10.32	6.25	11.59
	Dissatisfied	5.26	5.78	5.10
	Very dissatisfied	0.96	1.26	0.87
Confidentiality	Very satisfied	45.89	55.80	42.82
	Satisfied	43.66	38.21	45.36
	Fairly satisfied	8.20	4.83	9.24
	Dissatisfied	1.80	0.74	2.13
	Very dissatisfied	0.45	0.42	0.46
*Instrumental variable*				
Facility’s location	Very convenient	34.47	45.66	31.0
	Convenient	47.80	40.32	50.12
	Fairly convenient	11.54	10.69	11.81
	Not convenient	5.49	2.77	6.33
	Very inconvenient	0.70	0.56	0.74
*Control variable*				
Gender	Female = 1	83.72	76.33	86.01
Marital status	Married = 1	51.89	47.98	53.10
Employment status	Employed = 1	78.52	81.32	77.67
Residence	Urban = 1	44.67	76.95	48.63
Patient type	Outpatient = 1	82.89	82.88	82.93
Age	15 – 29	46.42	40.14	48.37
	30 – 44	42.25	47.64	40.58
	45 – 59	11.32	12.22	11.05
Level of education	Primary	14.65	7.88	16.75
	Secondary	56.64	63.9	54.39
	Higher	10.87	19.63	8.16
	Poorest	15.10	3.74	18.62
Wealth status	Poorer	15.81	6.63	18.65
	Middle	20.61	14.6	22.47
	Richer	22.49	27.77	20.86
	Richest	25.99	47.26	19.39
Mode of payment	Cash	30.46	45.06	25.94
	Insurance	62.73	47.56	67.43
	Co-payment	5.68	6.10	5.54
	Others	1.13	1.28	1.08
Type of illness/services	Family planning	4.75	2.03	5.59
	Maternal healthcare Services	29.24	14.50	33.81
	Fever	25.55	33.75	23.01
	Diarrhoea	2.54	2.50	2.55
	HIV/AIDS/STI	0.95	1.22	0.86
	High blood pressure	2.90	5.90	1.97
	Ear/nose/throat infection	1.14	1.50	1.03
	Diabetes	0.58	1.10	0.41
	Eye infection	0.89	0.64	0.96
	Check-up/preventive care	7.71	9.86	7.05
	Accident/injuries	3.54	4.15	3.36
	Child illness	4.99	1.62	6.03
	Own illness	7.93	11.80	6.73
	Others	7.31	9.43	6.66

Source for this and all subsequent tables: Author’s calculations from the 2014 GDHS individual data for respondents who visited/used health facilities six months before the survey. We accounted for the survey design using the sampling weights variable in the data. Results for the facility’s location are the observed results

**Fig. 1:**
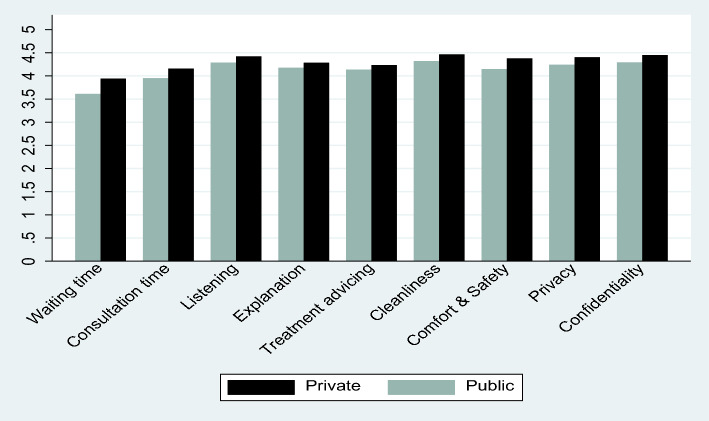
Mean scores of the healthcare services by a public and private facility (2014 Ghana DHS individual data). Note: Mean scores are based on the 5-point Likert from 1 = “Very dissatisfied” to 5 = “Very satisfied”. A high mean score indicates higher satisfaction with healthcare services, while lower mean scores suggest otherwise

Furthermore, the private facility users often indicated that they were “very satisfied”, whereas the public facility users often indicated they were “satisfied”. Also, the mean scores of the private facilities for all the healthcare services (quality indicators) are slightly above (higher) that of the public facilities, implying that, on average private facility users are a bit satisfied with the healthcare services than public users (see Fig. 1).

### First stage regression: the probability of using a private healthcare provider

The marginal effect estimates for the first-stage regression result are presented in Table 3. The results show that individuals who opine that the location of the private facility is “convenient”, “fairly convenient”, and “inconvenient” for them are respectively 5 pp, 7 pp, and 10 pp less likely to use private facilities compared to those who opine that the facility’s location is “very convenient” to them. Similarly, the probability of using private healthcare facilities increases as the location of the public facilities becomes very inconvenient for individuals. Furthermore, those from households in the middle, rich, and richest wealth categories are 7 pp, 1.6 pp and 2.3 pp more likely to use private facilities compared to those in the poorest category. We also found that females are 4 pp less likely to visit private facilities than men. Regarding the type of illness/service, we found that family planning, child illness and maternal healthcare patients are 18 pp, 13 pp and 21 pp less likely to visit private facilities compared to fever patients.

**Table 3:** First stage probit regression: Marginal effect results on the factors that influence the use of private healthcare facilities (from the 2014 Ghana DHS individual data)

Variables	Marginal effects	Standard errors
*Public facility location (ref. group; very convenient)*		
Convenient	0.02	0.02
Fairly convenient	0.03	0.03
Inconvenient	0.04	0.04
Very inconvenient	0.10	0.09
*Private facility location (ref. group; very convenient)*		
Convenient	− 0.05***	0.02
Fairly convenient	− 0.07***	0.02
Inconvenient	− 0.10***	0.03
Very inconvenient	− 0.01	0.10
Female	− 0.04*	0.02
Urban	0.04	0.03
Employed	0.02	0.02
Married	− 0.02	0.02
Outpatients	− 0.03	0.05
*Age (ref. group: 15–29)*		
30–44	0.04**	0.02
45–59	− 0.00	0.02
*Education (ref. group: no education)*		
Primary	− 0.03	0.03
Secondary	0.04	0.03
Higher	0.07*	0.04
*Wealth (ref. group: poorest)*		
Poorer	0.03	0.02
Middle	0.07***	0.03
Richer	0.16***	0.03
Richest	0.23***	0.04
*Mode of payment (ref. group cash)*		
Insurance	− 0.10***	0.02
Co−payment	− 0.09**	0.04
Others	− 0.04	0.08
*Type of illness/Services (ref. group fever)*		
Family planning	− 0.18***	0.03
Maternal healthcare services	− 0.13***	0.02
Child illness	− 0.21***	0.04
Log pseudolikelihood	− 1.895e + 09	
Wald ${\chi }^{2}$	440.84	
Prob > ${\chi }^{2}$	0.00	
Pseudo ${R}^{2}$	0.17	
Joint Significance ${\chi }^{2}$	22.39	
Prob > ${\chi }^{2}$	0.004	
Observations	4,323	

We accounted for clustering and sampling weights. For the type of illness/services, we report only those which were significant. *** *p* < 0.01, ** *p* < 0.05, * *p* < 0.1

### Tests of endogeneity

Prior to estimating the effect of facility ownership on quality, we tested the endogeneity of provider choice. The ordered probit regression model with endogenous binary regressor (IV results) produces a parameter that captures the correlation between the error terms in Eq. 1 (satisfaction of service equation) and Eq. 3 (provider choice equation). The endogeneity of the binary regressor is tested under the null hypothesis that there is no endogeneity (StataCorp, 2019). The results show that seven of the quality indicators are endogenous (waiting time, consultation time, listening, cleanliness, comfort and safety, confidentiality, and privacy). We found that the estimated correlation between the errors from the satisfaction of healthcare service equation and the errors from the provider choice equation for the seven quality indicators are significantly different from zero (0); hence, we rejected the null that the choice of a healthcare provider is exogenous. Also, all the estimates are positive; hence, we conclude that the unobserved factors that increase the chance of visiting a private healthcare facility tend to increase the chance of being satisfied with the healthcare services. We report the endogeneity test in Table 4.

**Table 4:** Second stage regression results (Oprobit and IV): Marginal effect of private ownership on the probability of different satisfaction levels on various dimensions of healthcare services (from the 2014 Ghana DHS individual data)

Private	Comfort and safety (N = 4324)		Consultation time (N = 4,247)		Confidentiality (N = 4324)		Treatment advice (N = 4324)
	Oprobit	IV	Oprobit	Oprobit	Oprobit	IV	Oprobit
Very satisfied	0.14***	0.46***	0.12***	0.48***	0.11***	0.42***	0.09***
	(0.02)	(0.05)	(0.02)	(0.06)	(0.02)	(0.05)	(0.03)
Satisfied	− 0.06***	− 0.27***	− 0.02***	− 0.20***	− 0.06***	− 0.26***	− 0.03***
	(0.01)	(0.03)	(0.01)	(0.03)	(0.01)	(0.04)	(0.01)
Fairly satisfied	− 0.05***	− 0.13***	− 0.05***	− 0.15***	− 0.04***	− 0.11***	− 0.03***
	(0.01)	(0.02)	(0.01)	(0.01)	(0.01)	(0.02)	(0.01)
Dissatisfied	− 0.02***	− 0.05***	− 0.03***	− 0.07***	− 0.01***	− 0.04***	− 0.02***
	(0.00)	(0.01)	(0.01)	(0.01)	(0.00)	(0.01)	(0.01)
Very dissatisfied	− 0.01***	− 0.02**	− 0.02***	− 0.06***	− 0.00***	− 0.02*	− 0.01***
	(0.00)	(0.01)	(0.00)	(0.02)	(0.00)	(0.01)	(0.00)
Log pseudolikelihood	− 4.266e + 09	− 6.155e + 09	− 4.810e + 09	− 6.675e + 09	− 4.156e + 09	− 6.046e + 09	− 4.755e + 09
Wald ${\chi }^{2}$	177.73	260.31	140.16	257.01	156.54	225.53	143.41
Prob > ${\chi }^{2}$	0.00	0.00	0.00	0.00	0.00	0.00	0.00
Corr (e.Private, e.care services)		0.56***		0.59***		0.54***	

Private	Cleanliness (N = 4324)		Waiting time (N = 4,314)		Listening (N = 4,323)		Privacy (N = 4324)		Explanation(N = 4324)
	Oprobit	IV	Oprobit	IV	Oprobit	IV	Oprobit	IV	Oprobit
Very satisfied	0.12***	0.42***	0.14***	0.42***	0.11***	0.41***	0.14***	0.46***	0.08***
	(0.02)	(0.07)	(0.02)	(0.06)	(0.02)	(0.04)	(0.02)	(0.02)	(0.03)
Satisfied	− 0.07***	− 0.28***	0.01***	− 0.08***	− 0.07***	− 0.27***	− 0.07***	− 0.27***	− 0.03***
	(0.01)	(0.01)	(0.00)	(0.03)	(0.01)	(0.02)	(0.01)	(0.04)	(0.01)
Fairly satisfied	− 0.03***	− 0.10***	− 0.05***	− 0.12***	− 0.03***	− 0.09***	− 0.04***	− 0.11***	− 0.03***
	(0.01)	(0.02)	(0.01)	(0.01)	(0.01)	(0.02)	(0.01)	(0.02)	(0.01)
Dissatisfied	− 0.01***	− 0.03***	− 0.06***	− 0.12***	− 0.01***	− 0.04***	− 0.02***	− 0.05***	− 0.02***
	(0.00)	(0.01)	(0.01)	(0.02)	(0.00)	(0.01)	(0.00)	(0.02)	(0.01)
Very dissatisfied	− 0.00**	− 0.01*	− 0.05***	− 0.10***	− 0.00**	− 0.01	− 0.01***	− 0.03	− 0.00**
	(0.00)	(0.01)	(0.01)	(0.02)	(0.00)	(0.01)	(0.00)	(0.02)	(0.00)
Log pseudolikelihood	− 3.900e + 09	− 5.790e + 09	− 5.740e + 09	− 7.628e + 09	− 4.045e + 09	− 5.936e + 09	− 4.393e + 09	− 6.279e + 09	− 4.549e + 09
Wald ${\chi }^{2}$	194.28	282.07	146.47	160.02	134.27	217.47	152.08	248.36	123.59
Prob > ${\chi }^{2}$	0.00	0.00	0.00	0.00	0.00	0.00	0.00	0.00	0.00
Corr (e.Private, e.care services)		0.57***		0.47***		0.52***		0.58***	

Survey design (sampling weights and clustering) were accounted for in all estimations. Standard errors are in parentheses

We control for individual characteristics (age, gender, education, wealth, residence, employment status, marital status, patient type, type of illness/service and mode of payment). *** *p* < 0.01, ** *p* < 0.05, * *p* < 0.1

### Validity of the instrument

After confirming the endogeneity of the quality indicators, we tested the validity of the instrumental variable based on two assumptions. First, the facility’s location should be correlated with the choice of the facility. The stronger the correlation, the stronger the identification (Cameron & Trivedi, 2009, p. 175). We test this assumption by performing a chi-squared test to determine the joint significance of the instruments (convenience of the facility’s location) in predicting the probability of visiting a public or private healthcare facility. The chi-squared statistic is 22.39 and is statistically significant at a 1% significant level (*p*-value = 0.004), which signifies that the convenience of the facility’s location is significantly corrected with the probability of visiting a public or private healthcare facility. The chi-squared result is presented in Table 3.

The second assumption is that the instrument variable should be uncorrelated with the outcome variable (quality of the healthcare services) except through its effect on the endogenous variable (healthcare providers). This assumption cannot be directly tested because of the role of unobserved factors (Wehby et al., 2009). All things being equal, we expect that patients would prefer to use healthcare facilities where it is convenient for them. Moreover, previous studies used a similar variable as instruments and proved its validity using the over-identification test (Lien et al., 2008; Moscelli et al., 2018).

### Second stage regression (Oprobit and IV): The effect of facility ownership on quality

Table 4 presents the marginal effect estimates of healthcare facility ownership on the quality of healthcare services. The results show the effect of visiting a private healthcare facility on the probability of being very satisfied/dissatisfied with the healthcare services. The IV estimate accounts for the endogeneity of healthcare facility ownership status, while the oprobit estimate does not. We did not account for endogeneity for the two quality indicators (treatment advice and explanation) since the results from the endogeneity test revealed that the two variables are exogenous.

In line with the descriptive statistics, both the IV and oprobit results show that for all the quality indicators, private facility users are more likely to be “very satisfied” with the healthcare services they receive than public users. The IV estimates on “waiting time”, “consultation time”, “listening”, “cleanliness”, “comfort and safety”, “confidentiality”, and “privacy” show that private users are 42 pp, 48 pp, 41 pp, 42 pp, 46 pp, 42 pp, and 46 pp respectively more likely to be very satisfied. Likewise, the oprobite estimates on “waiting time”, “consultation time”, “listening”, “cleanliness”, “comfort and safety”, “confidentiality”, and “privacy” show that private users are, respectively 14 pp, 12 pp, 11 pp, 12 pp, 14 pp, 11 pp, and 14 pp more likely to be very satisfied. Furthermore, both the IV and oprobit estimates show that private facility users are less likely to be dissatisfied with their healthcare services.

In addition, we found that the IV estimates are larger than the oprobit estimates. For instance, the oprobit results on “waiting time”, “consultation time”, “listening”, “cleanliness”, “comfort and safety”, “confidentiality”, and “privacy” shows that private users are, respectively 14 pp, 12 pp, 11 pp, 12 pp, 14 pp, 11 pp, and 14 pp more likely to be very satisfied, however, the IV estimates show that the private users are 42 pp, 48 pp, 41 pp, 42 pp, 46 pp, 42 pp, and 46 pp respectively more likely to be very satisfied.

## Discussion

We investigate whether there are differences in healthcare quality between public and private healthcare providers using the 2014 DHS data from Ghana. Results from the study revealed that private facility users are more likely to be “very satisfied” with the healthcare services they receive than public users. Similarly, private facility users are less likely to be dissatisfied with their healthcare services. These results suggest that private healthcare facilities provide better service than public facilities. Our findings are consistent with those of similar studies from Ghana and other sub-Saharan African countries that also reported that private healthcare providers provide better services than public healthcare providers (Adongo et al., 2021; Alhassan et al., 2016; Anabila et al., 2019; Fall, 2017; Owusu Kwateng et al., 2017).

A couple of reasons may explain why respondents are more satisfied with healthcare services at private facilities than in public facilities. It may be that private providers often meet the demands/needs of their patients (Agyemang-Duah et al., 2020), unlike public providers who are often criticised for not responding to the needs/demands of their patients (Adongo et al., 2021; Anabila et al., 2019). Furthermore, due to the private sector’s profit motive, they may treat their patients with enough courtesy. The private providers know that if they are rude to patients, they may not use their facilities anymore, affecting their profit level. As such, the workers in the private facilities are often more friendly and easy to interact with than those in the public facilities (Agyemang-Duah et al., 2020; Anabila et al., 2019; Owusu Kwateng et al., 2019). In addition, because the cost of healthcare in the public sector is cheaper compared to the private sector, they (public providers) are able to attract more patients (Agyemang-Duah et al., 2020). A large number of patients may lead to longer hours of waiting time at public facilities. This may explain why public users are less satisfied with “waiting time” than private users (Alhassan et al., 2016; Anabila et al., 2019). It could also be that because of the large number of patients, the healthcare personnel do not spend enough time with patients to serve other patients. Therefore, the patients may not be satisfied with the quality of consultation time at public facilities.

Furthermore, the study revealed that the IV estimates are larger than the “oprobit” estimates thus suggesting that estimating the effect of healthcare facility ownership on the quality of healthcare services without accounting for endogeneity in provider choice underestimates the results. This may be because private users often demand a higher quality healthcare service due to the high cost of healthcare service at private healthcare facilities. Hence, they may rate the quality of healthcare service as “not very satisfying” if the healthcare service is slightly below what they expect. The public users, on the other hand, may expect an average health service quality because they do not incur higher medical costs like the private users. However, if the public users compare the quality of healthcare services they receive to that of the private users, they (public users) would rate the quality of service given to private users as “very satisfying”. Also, if the private users compare the quality of healthcare services they receive to that of the public users, they (private users) would rate the quality of service given to public users as “very dissatisfying”. Our findings are similar to that of Moscone et al. (2020) in Italy—their OLS estimates showed that private hospitals have a lower mortality rate for AMI patients by 2.7 pp and a readmission rate for hip replacement patients by 0.24 pp; however, the IV estimates revealed that private hospitals have lower mortality rate for AMI patients by 3.5 pp and a readmission rate for hip replacement patients by 3.6 pp.

### Further analysis (rural and urban sample)

We further divided the sample by rural and urban to analyse the effect of facility ownership on healthcare quality by residence. The results are provided in Table 5. It shows that urban residents who use private healthcare facilities are more likely to be “very satisfied” with the healthcare services they receive. In contrast, rural residents who use private facilities are less likely to be “very satisfied” with the healthcare services they receive. For instance, the results on “cleanliness” indicate that compared to public facilities, urban residents who used private facilities are 52 pp more likely to be “very satisfied” with the cleanliness of the facility. However, rural residents who used private facilities are 33 pp less likely to be “very satisfied” with the cleanliness of the facility. Our findings suggest that urban residents perceive that private healthcare facilities provide better healthcare services than public facilities, whereas rural residents perceive otherwise. This may be because the private provider reduces the quality of services provided to rural areas. The private providers know that people in rural areas may not be able to afford the medical cost of “standard services”; hence, they may reduce the “standard of service” in the rural areas to match the service cost. It could also be that rural residents’ health demands are less than urban residents. Since the private providers are profit-oriented, they are more likely to channel their resources to where they may be needed most and can get them higher profits, often in the urban areas.

**Table 5:** Marginal effect of private ownership on the probability of different satisfaction levels on various dimensions of healthcare services by place of residence (Rural–Urban) (from the 2014 Ghana DHS individual data)

Private Facility	Comfort and Safety		Consultation time		Confidentiality		Treatment advice	
	Urban	Rural	Urban	Rural	Urban	Rural	Urban	Rural
Very satisfied	0.54***	− 0.22***	0.56***	− 0.21***	0.51***	− 0.32***	0.52***	− 0.30***
	(0.02)	(0.08)	(0.04)	(0.04)	(0.02)	(0.04)	(0.03)	(0.05)
Satisfied	− 0.23***	0.01	− 0.18***	− 0.10*	− 0.25***	0.02	− 0.17***	− 0.03
	(0.01)	(0.04)	(0.01)	(0.06)	(0.02)	(0.04)	(0.01)	(0.04)
Fairly satisfied	− 0.14***	0.12**	− 0.14***	0.14***	− 0.13***	0.17***	− 0.10***	0.14***
	(0.01)	(0.06)	(0.01)	(0.03)	(0.01)	(0.03)	(0.01)	(0.03)
Dissatisfied	− 0.08***	0.07	− 0.10***	0.09***	− 0.07***	0.08**	− 0.14***	0.12***
	(0.01)	(0.05)	(0.01)	(0.03)	(0.02)	(0.03)	(0.01)	(0.04)
Very dissatisfied	− 0.09***	0.01	− 0.14***	0.08*	− 0.06***	0.04*	− 0.11***	0.05*
	(0.02)	(0.01)	(0.03)	(0.05)	(0.01)	(0.02)	(0.03)	(0.03)
Log pseudolikelihood	− 3.630e + 09	− 2.405e + 09	− 3.971e + 09	− 2.591e + 09	− 3.552e + 09	− 2.380e + 09	− 3.966e + 09	− 2.542e + 09
Wald ${\chi }^{2}$	1072.84	126.75	635.29	69.08	800.87	161.95	543.05	163.48
Prob > ${\chi }^{2}$	0.00	0.00	0.00	0.00	0.00	0.00	0.00	0.00
Corr(e.Private, e.care services)	0.83***	− 0.59***	0.79***	− 0.59***	0.77***	− 0.64***	0.83***	− 0.62***

Private Facility	Cleanliness		Waiting time		Listening		Privacy		Explanation	
	Urban	Rural	Urban	Rural	Urban	Rural	Urban	Rural	Urban	Rural
Very satisfied	0.52***	− 0.33***	0.56***	− 0.23***	0.49***	− 0.35***	0.56***	− 0.30***	0.49***	− 0.33***
	(0.02)	(0.06)	(0.06)	(0.02)	(0.03)	(0.05)	(0.02)	(0.04)	(0.02)	(0.05)
Satisfied	− 0.27***	0.07**	− 0.09***	− 0.19***	− 0.27***	0.04	− 0.22***	0.00	− 0.20***	− 0.02
	(0.01)	(0.03)	(0.01)	(0.04)	(0.01)	(0.04)	(0.02)	(0.04)	(0.01)	(0.05)
Fairly satisfied	− 0.12***	0.17***	− 0.10***	0.05***	− 0.11***	0.17***	− 0.11***	0.18***	− 0.11***	0.16***
	(0.01)	(0.05)	(0.01)	(0.01)	(0.01)	(0.03)	(0.01)	(0.04)	(0.01)	(0.03)
Dissatisfied	− 0.06***	0.07**	− 0.15***	0.14***	− 0.07***	0.10**	− 0.08***	0.08**	− 0.12***	0.12***
	(0.01)	(0.03)	(0.01)	(0.02)	(0.01)	(0.04)	(0.02)	(0.03)	(0.01)	(0.04)
Very dissatisfied	− 0.06***	0.01	− 0.22***	0.22***	− 0.05***	0.03	− 0.14***	0.03	− 0.06***	0.06*
	(0.02)	(0.01)	(0.02)	(0.06)	(0.02)	(0.02)	(0.04)	(0.02)	(0.02)	(0.03)
Log pseudolikelihood	− 3.387e + 09	− 2.307e + 09	− 4.437e + 09	− 3.067e + 09	− 3.523e + 09	− 2.308e + 09	− 3.728e + 09	− 2.391e + 09	− 3.843e + 09	− 2.474e + 09
Wald ${\chi }^{2}$	707.75	142.39	522.86	130.68	412.28	157.01	1058.52	155.93	317.08	153.22
Prob > ${\chi }^{2}$	0.00	0.00	0.00	0.00	0.00	0.00	0.00	0.00	0.00	0.00
Corr(e.Private, e.care services)	0.77***	− 0.57**	0.78***	− 0.69***	0.68***	− 0.66***	0.88***	− 0.66***	0.71***	− 0.63***

Survey design (sampling weights and clustering) were accounted for in all estimations. Standard errors are in parentheses

We control for individual characteristics (age, gender, education, wealth, employment status, marital status, patient type, type of illness/service and mode of payment). *** *p* < 0.01, ** *p* < 0.05, * *p* < 0.1

## Conclusion

The study set out to investigate whether healthcare facility ownership has an effect on quality using data from the 2014 GDHS.

The study revealed that for all the quality indicators, private facility users are more likely to be very satisfied with the services they receive than public users, thus implying that private healthcare facilities provide better service than public facilities. Furthermore, we found that a patient’s choice of a healthcare provider is endogenous; hence estimating the effect of healthcare facility ownership on healthcare services quality without accounting for endogeneity in provider choice underestimates the results. In addition, we found that urban residents perceive that private healthcare facilities provide better healthcare services than public facilities, whereas rural residents perceive otherwise.

The study’s findings have some policy implications for Ghana and other sub-Saharan African countries moving towards expanding the private health sector’s role. Given that our findings suggest that private facilities provide better healthcare services, we recommend that the government consider encouraging the private sector to enter the healthcare market. Alternatively, the government may consider instituting strategies/policies that will target improving the competence, efforts, and attitudes of the public healthcare providers to help them improve the quality of services (WHO, 2000). Such strategies may include training (pre-service or in-service), protocols and guidelines, supervision, audit and feedback (Dayal & Hort, 2015). In addition, the government could consider motivating the public providers with materials or moral incentives to help them improve the quality of services provided to patients.

### Limitations

Although the study provides vital information for policy implementation, it also has a few limitations. Primarily, private providers can be for-profit or not-for-profit, and the resulting differences in incentives might affect the quality of services they provide. In this study, however, we could not separate the private providers. This is because the data we used did not specify whether the private facilities used by the individuals are for-profit or not-for-profit. Another limitation is the subjective nature of the quality indicators. The challenge with subjective data is that patients’ opinions about quality healthcare may differ from experts’ (providers’) opinions. Moreover, some patients may likely rate the quality of healthcare services as good if they have family or friends working in the facility they visited. Thus, we suppose our results could improve if we included the opinions of healthcare experts or an objective measure of healthcare quality in the study. However, we could not include these variables due to data limitations. Also, the Ministry of Health in Ghana broadly categorizes health facilities into four ownership categories; public (government), private, quasi-government, and faith-based or missionary health facilities (Ministry of Health—Ghana, 2012); however, this study is limited to only public and private healthcare facility due to unavailability of data for the quasi-government, and faith-based facilities. Furthermore, a mixed-method study could help in understanding the reasons behind the study’s findings. However, we were unable to do a mixed-method study due to data limitations.

## Data Availability

The data is owned by a third party – Demographic and Health Survey (DHS). The data are publicly available on the DHS website (www.dhsprogram.com). Interested parties can request for the data from DHS.

## Code availability

Code for data cleaning and analysis is available upon request.
